# Laparoscopic treatment of ureteroduodenal fistula: Case report, technique, and review

**DOI:** 10.1016/j.eucr.2025.103331

**Published:** 2025-12-30

**Authors:** Alexandre Saboia Leitão, Marllon Rodrigues Ferreira, Rommel Prata Regadas, Ricardo Reges Maia de Oliveira, Gabriel Silva Lima

**Affiliations:** Department of Surgery, Universidade Federal Do Ceará, Fortaleza, CE, Brazil

**Keywords:** Ureteral fistula, Duodenum/surgery, Laparoscopy, Ureterolithiasis/complications

## Abstract

Ureteroduodenal fistula is extremely rare, with fewer than 20 reported cases. We describe a 50-year-old woman with recurrent flank pain and hematuria whose imaging showed proximal ureteral hydronephrosis, a 2-cm stone, and a migrated metallic foreign body. During laparoscopic ureterolithotomy, dense adhesions revealed a fistula caused by transduodenal needle penetration with associated stone formation. Laparoscopic removal of the stone and foreign body, duodenal repair, and ureteral suturing with stent placement were successfully performed. The patient recovered uneventfully and remained asymptomatic at 6 months. This represents the first laparoscopic management of ureteroduodenal fistula, highlighting a safe, kidney-sparing alternative to open surgery.

## 1 Introduction

Ureteroduodenal fistulas are exceedingly rare, with fewer than 20 cases reported in the literature. The primary goal of management is definitive closure of the fistula with restoration of urinary and intestinal integrity. Conventional treatment historically involved laparotomy for fistula repair. Although laparoscopic techniques have been described for other urinary–intestinal fistulas, such as colovesical fistulas, this is the first reported case of a ureteroduodenal fistula to be successfully managed through a laparoscopic approach. Here, we describe a case of ureteroduodenal fistula secondary to foreign body ingestion (a needle), which in turn led to proximal ureteral stone formation and was definitively treated using laparoscopy.

## 2 Case report

A 50-year-old woman, a dressmaker, presented with a 6-month history of recurrent right lumbar pain and episodes of macroscopic hematuria. She also reported uncharacteristic abdominal pain and dyspepsia. Physical examination revealed no significant findings.

**Fig. 1:**
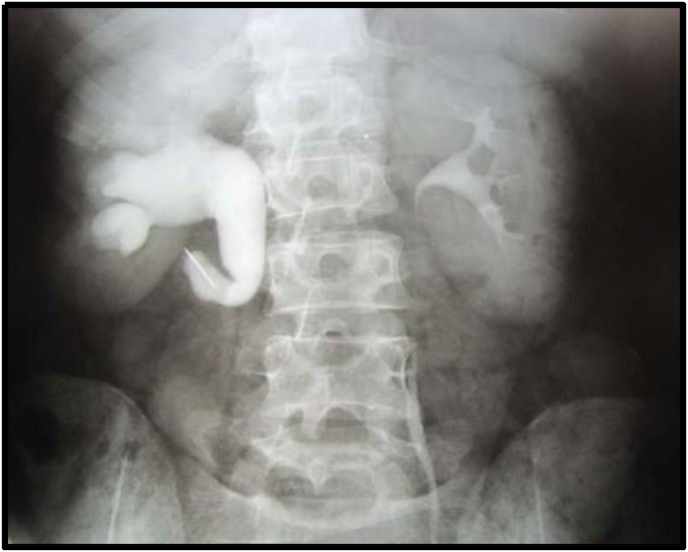
Excretory urography showing right-sided hydronephrosis, along with a calculus and a metallic foreign body in the right upper ureter.

Abdominal ultrasonography demonstrated right proximal ureteral hydronephrosis and a 2-cm upper ureteral calculus. Excretory urography confirmed dilation of the right excretory system and revealed that the calculus was located over a metallic foreign body in the upper right ureter (Fig. 1). Given the stone size and location, laparoscopic ureterolithotomy was indicated.

## 3 Laparoscopic technique

The patient was placed in the left lateral decubitus position under general anesthesia. She was secured to the operating table with adhesive tape to maintain stability. The urologic surgeon stood on the patient's left side, accompanied by the assistant and cameraman.

A 10-mm trocar was inserted at the lateral border of the rectus abdominis muscle following the Hasson technique. A CO_2_ pneumoperitoneum was established and maintained at 15 mmHg. A 30-degree laparoscopic camera was introduced, followed by placement of two additional ports (10 mm and 5 mm) in the right upper quadrant for instrumentation.

Mobilization of the right colon revealed an intense inflammatory process involving the ureter and duodenum. After sharp and blunt dissection of the adhesions, the ureteroduodenal fistula was identified (Fig. 2A, Fig. 2BA and B). Upon further separation of the duodenum from the ureter, a metallic needle penetrating through the duodenum and contributing to stone formation was discovered (Fig. 3A). The duodenum was closed with a one-layer interrupted suture (Fig. 3B). The ureter was then longitudinally incised, allowing removal of both the stone and the needle (Fig. 3B, Fig. 4AA).

**Fig. 2A:**
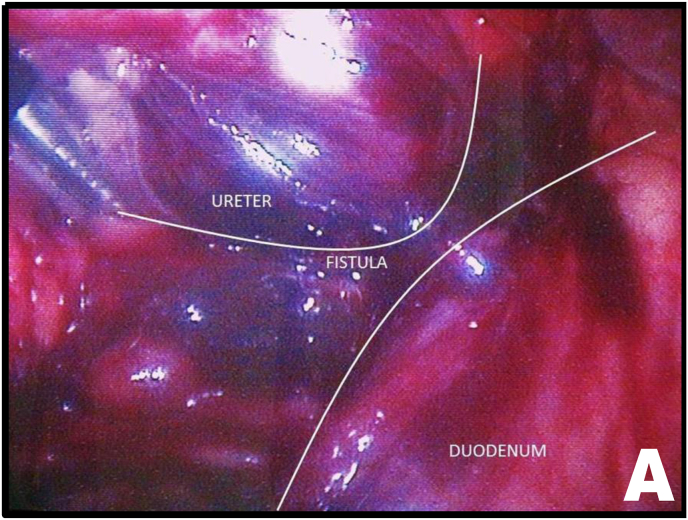
Ureteroduodenal fistula identified.

**Fig. 2B:**
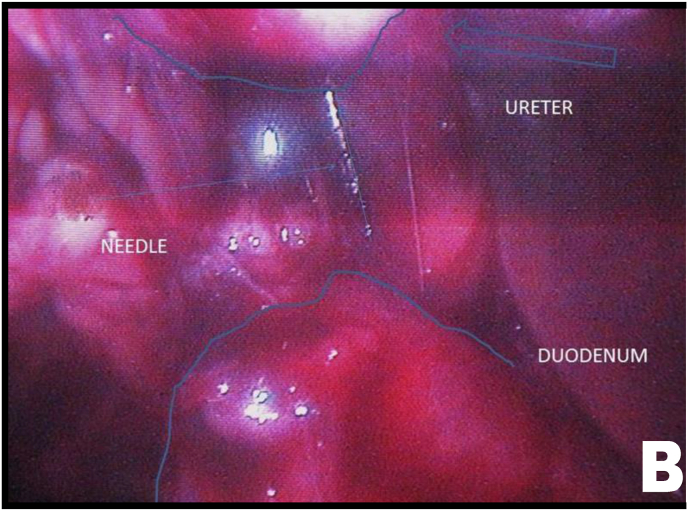
Ureteroduodenal fistula and metallic foreign body (needle).

**Fig. 3A:**
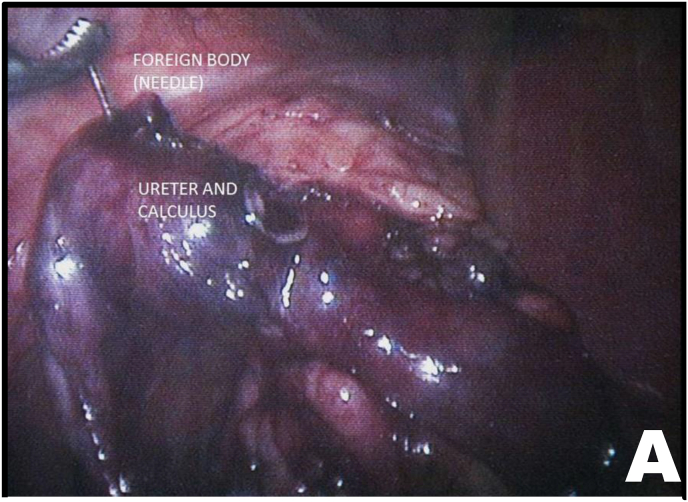
Needle penetrating the ureter and associated ureteral calculus.

**Fig. 3B:**
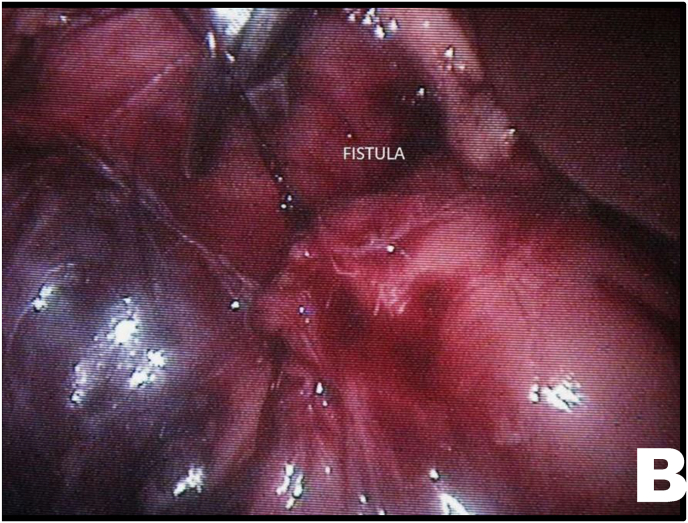
Duodenal fistula closure.

**Fig. 4A:**
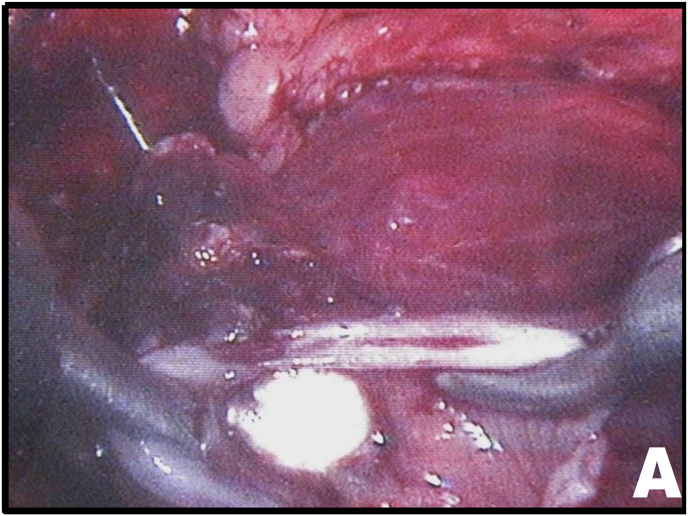
Excision of the ureteral stone and needle.

A 6F double-J ureteral stent was placed, and the ureter was closed with a continuous running suture using 4-0 Vicryl (Fig. 4B). After stone and foreign body removal (Fig. 5A), a 4.8 Fr tubular suction drain was positioned, and the ports were closed.

**Fig. 4B:**
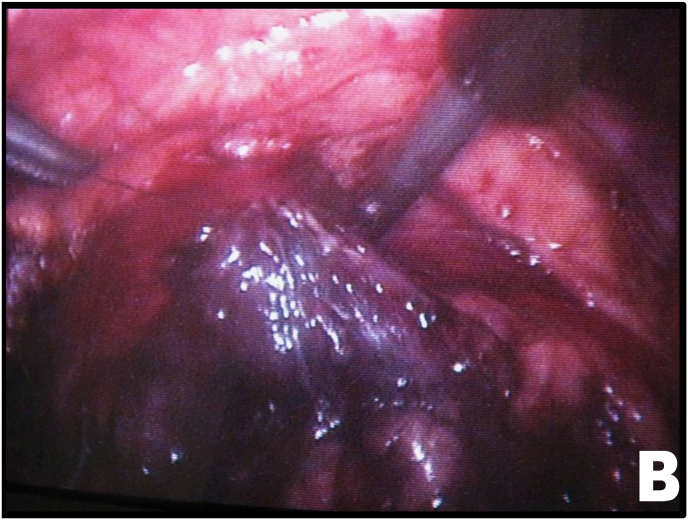
Continuous suture of the ureteral incision.

**Fig. 5A:**
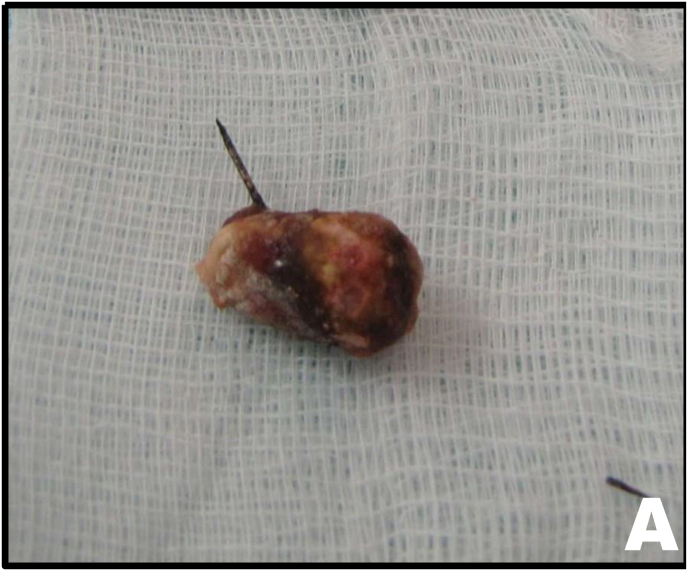
Ureteral stone and needle.

Total operative time was 2 hours and 15 minutes (135 minutes). The postoperative period was uneventful. The patient remained fasting for 72 hours and resumed oral intake on the third postoperative day, with no evidence of leakage. The drain was removed on postoperative day 4, and she was discharged in good condition. The ureteral stent was removed 6 weeks later.

Follow-up abdominal CT demonstrated resolution of hydronephrosis (Fig. 5B). At 6 months, the patient remained asymptomatic; a control CT scan revealed an unrelated mass in the right kidney.

**Fig. 5B:**
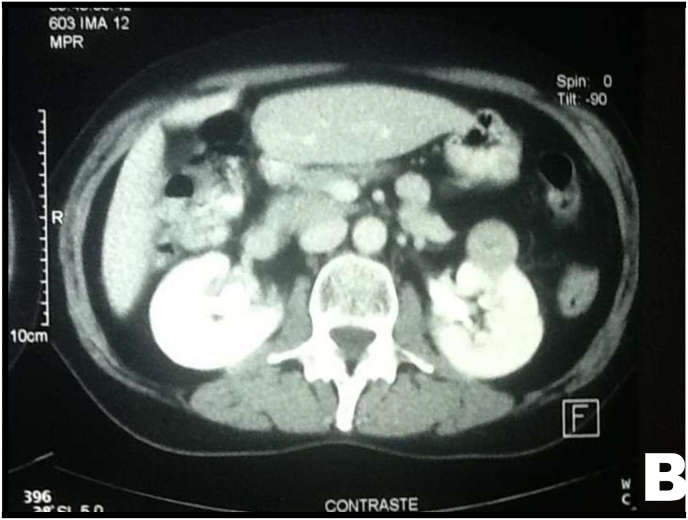
Abdominal CT showing absence of right-sided hydronephrosis and the presence of a renal mass in the right kidney.

## 4 Discussion

Ureteroduodenal fistula is an exceptionally rare condition, with fewer than 20 cases reported to date, and its diagnosis remains particularly difficult due to the nonspecific clinical presentation and heterogeneous etiologies. The first case was documented by Davis in 1918,^1^ and the second only in 1969 by Desleaver et al.^2^ Historically, these patients were treated with exploratory laparotomy, nephrectomy, and duodenal closure, and both early reports were attributed to chronic infection. Other reported etiologies include abdominal trauma, gunshot wounds with abscess formation, ureteral calculi, and perforated duodenal ulcer.^3^, ^4^, ^5^, ^6^ The present case represents the second attributed to foreign body ingestion and the fourth originating primarily from the duodenum. Roberts et al. described a case of duodenal perforation caused by a foreign body without stone formation,^7^ whereas Wu reported a ureteroduodenal fistula secondary to a perforated duodenal ulcer.^8^

The present case is unique, as it represents the first known instance of a ureteroduodenal fistula treated laparoscopically, allowing simultaneous removal of the foreign body and calculus and complete correction of the fistula, demonstrating that kidney preservation is feasible without resorting to laparotomy. Minimally invasive techniques have been increasingly adopted in urologic fistula repair, with favorable outcomes reported in other contexts as well.^9^ Similarly, Bachelier et al. reported successful laparoscopic repair of a nephrocolic fistula, reinforcing the applicability of minimally invasive techniques and their benefits, such as reduced invasiveness and faster postoperative recovery.^10^

Typical symptoms of ureteroduodenal fistula include fever, flank pain, and pneumaturia. Hematuria and pyuria are common early findings, and recurrent urinary tract infections frequently prompt imaging studies that lead to diagnosis. In this case, foreign body migration and perforation of the duodenum and ureter directly contributed to calculus formation, a mechanism not previously described in the literature. Given the stone size (>1 cm in the proximal ureter), laparoscopic ureterolithotomy was appropriate and aligns with established contemporary indications for the management of ureteral stones.^11^ The fistula was discovered intraoperatively, as gastrointestinal manifestations were absent.

Management of ureteroduodenal fistula remains challenging and is not standardized, largely due to its rarity and diverse presentations. When renal function is preserved, primary duodenal closure and direct ureteral repair with stenting are suitable options. After stone extraction, placement of a temporary double-J ureteral catheter is typically recommended to ensure drainage and healing. In our patient, all steps—fistula closure, foreign body and stone removal, and temporary ureteral drainage—were successfully accomplished through a minimally invasive approach.

In contrast, since this report, only three additional cases have been documented. Patel et al. described a 29-year-old man who developed a fistula between the proximal right ureter and the third portion of the duodenum after inadequate postoperative follow-up following open ureterolithotomy and stent placement. The patient underwent open surgical exploration with excision of the fistulous tract, two-layer duodenal closure, and ureteral suturing over a new double-J stent.^12^

Makary et al. reported a 66-year-old woman who developed a fistula between the proximal right ureter and the second portion of the duodenum after prior endoscopic treatment of a ureteropelvic junction stone in a kidney affected by xanthogranulomatous pyelonephritis. She underwent emergent nephrectomy, initially attempted laparoscopically but converted to open surgery due to dense adhesions and bleeding.^13^

Tan et al. described a 45-year-old man with prior surgery for ureteral stone and recent nephrectomy for hydronephrosis with infection and ureteral dilation, who developed a fistula between the upper right ureter and the junction of the second and third duodenal portions. Following unsuccessful conservative treatment, revisional surgery with excision of the fistulous tract and two-layer duodenal closure was performed.^14^

These recent cases illustrate that all patients developed ureteroduodenal fistula secondary to pre-existing urologic conditions and were treated with open surgical techniques, highlighting the uniqueness and significance of the laparoscopic approach employed in our case.

## 5 Conclusion

Ureteroduodenal fistula remains an exceedingly rare and diagnostically challenging condition, requiring meticulous clinical evaluation and appropriate imaging for detection. This case underscores laparoscopy as a technically significant and valuable alternative to conventional open surgery, offering effective fistula repair, renal preservation, and rapid postoperative recovery—even in anatomically complex scenarios. These advantages are particularly relevant for patients with multiple comorbidities or elevated surgical risk.

Given the scarcity of similar reports, this case contributes meaningfully to the growing body of evidence supporting minimally invasive management of urinodigestive fistulas. Additional case reports and long-term follow-up are needed to strengthen the role of laparoscopy as a preferred therapeutic option for these rare and complex presentations.

## CRediT authorship contribution statement

**Alexandre Saboia Leitão:** Conceptualization, Data curation, Investigation, Methodology, Writing – original draft, Writing – review & editing. **Marllon Rodrigues Ferreira:** Data curation, Investigation, Writing – original draft, Writing – review & editing. **Rommel Prata Regadas:** Project administration, Supervision, Validation, Visualization. **Ricardo Reges Maia de Oliveira:** Project administration, Supervision, Visualization. **Gabriel Silva Lima:** Data curation, Investigation, Visualization, Writing – original draft.

## Patient consent

Written informed consent was obtained from the patient for publication of this case report and accompanying images.

## AI-assisted technologies statement

Durante a preparação deste manuscrito, os autores utilizaram o ChatGPT (OpenAI) para auxiliar na revisão linguística. Após a utilização dessa ferramenta, os autores revisaram e editaram o conteúdo conforme necessário e assumem total responsabilidade pela versão final do manuscrito.

## Declaration of competing interest

The authors declare no conflicts of interest.
